# Optimizing environmental viral surveillance: bovine serum albumin increases RT-qPCR sensitivity for high pathogenicity avian influenza H5Nx virus detection from dust samples

**DOI:** 10.1128/spectrum.03055-23

**Published:** 2023-11-20

**Authors:** Pierre Bessière, Brandon Hayes, Fabien Filaire, Laetitia Lèbre, Timothée Vergne, Matthieu Pinson, Guillaume Croville, Jean-Luc Guérin

**Affiliations:** 1 IHAP, Université de Toulouse, INRAE, ENVT, Toulouse, France; 2 THESEO France, LanXess Biosecurity, LanXess Group, Laval, France; 3 Labovet, Challans, France; Utrecht Institute for Pharmaceutical Sciences, Utrecht University, Utrecht, the Netherlands

**Keywords:** influenza, virology, avian viruses

## Abstract

The latest outbreaks of high pathogenicity avian influenza viruses (HPAIVs) were the most widespread ever seen in Europe, entailing considerable economic costs and raising public health concerns. Virological surveillance protocols involve reverse transcription quantitative polymerase chain reactions (RT-qPCR) from tracheal and cloacal swabs, which are laborious to perform on a large scale and require special skills. Environmental sampling, and especially dust collection, may represent a relevant alternative, as it is cheap, non-invasive for animals, simpler, and quicker to carry out. The main drawback of dust samples is that they may contain high amounts of organic substances that can inhibit RT-qPCR reactions. Bovine serum albumin (BSA) is a molecule known to facilitate DNA polymerization in the presence of numerous inhibitors, including those from feces, litter, or food. We tested its use on dust samples collected on 107 farms localized in areas affected by epizootics of clade 2.3.4.4b HPAIV H5N1. We used a latent class modeling approach to compare the performance of three detection protocols: (i) BSA addition to the RT-qPCR reaction mix, (ii) dilution of template RNA, and (iii) a control protocol. Our results indicate that BSA addition to the RT-qPCR reaction mix improved the sensitivity of the method, by neutralizing inhibitors’ effect. Indeed, for hemagglutinin (HA) or matrix (M) RNA detection, the sensitivity of the BSA protocol was the highest, followed by that of the control protocol, and that of the dilution protocol. Our results suggest that the use of BSA could be routinely implemented in HPAIV dust monitoring RT-qPCR protocols.

**IMPORTANCE:**

With the circulation of high pathogenicity avian influenza viruses having intensified considerably in recent years, the European Union is considering the vaccination of farmed birds. A prerequisite for this vaccination is the implementation of drastic surveillance protocols. Environmental sampling is a relevant alternative to animal sampling. However, environmental samples often contain inhibitory compounds in large enough quantities to inhibit RT-qPCR reactions. As bovine serum albumin is a molecule used in many fields to overcome this inhibitory effect, we tested its use on dust samples from poultry farms in areas heavily affected by HPAIV epizootics. Our results show that its use significantly increases the sensitivity of the method.

## INTRODUCTION

High pathogenicity avian influenza viruses (HPAIVs) have become a major threat to the poultry industry and wild bird populations (1, 2). These viruses can replicate systemically in birds and are known to be excreted in respiratory secretions and feces (1, 3). Poultry farms generate considerable quantities of dust, both from the environment (especially litter and feed) and from the animals themselves (especially skin and feathers) (4, 5). This dust is known to carry infectious viral particles within the infected farm (5), and sometimes over longer distances (6, 7).

The number of epizootics caused by HPAIV is increasing worldwide (8). Their surveillance involves tracheal or cloacal swab-based sampling, which is laborious and needs technical skills that make its application on a massive scale challenging. Environmental sampling, and especially dust sampling, maybe a relevant alternative and could play a role of major importance in monitoring and controlling the occurrence of epizootics (5). These samples are usually taken using dry wipes, rubbed into the surface of walls and farm equipment to collect dust. They are easier to perform than tracheal and cloacal swabs, as they do not require any special skills, and the sensitivity of this sampling method is not significantly different from that of swabs (5). Finally, they are inexpensive and non-invasive, which makes them ideally suited to large-scale surveillance protocols, now being considered in Europe following the introduction of vaccination against H5Nx HPAIVs (9).

The main drawback of dust samples is that they often contain inhibitory substances in sufficient amounts to disrupt reverse transcription quantitative polymerase chain reactions (RT-qPCR) or cause fluorescence inhibition (10, 11). PCR inhibitors may be present in various concentrations in many samples, regardless of their origin, but organic compounds present in the soil or resulting from digestion, like humic acid or tannic acid, which are known to inhibit RT-qPCR reactions, can frequently be found on dust samples (11, 12). Optimizing RT-qPCR protocols is therefore of major interest. Bovine serum albumin (BSA) is one of the most widely used molecules for facilitating DNA polymerization in the presence of organic inhibitors substances, whether in stool samples, forensic medicine, food hygiene, or veterinary medicine (12 – 16). However, to the best of our knowledge, its use has never been tested in dust samples taken from poultry farms for HPAIVs surveillance protocols.

We took dust samples from 107 poultry houses at very high risk of HPAI to determine how the addition of BSA to the RT-qPCR mix would affect sensibility and sensitivity. Three protocols were compared: a control protocol, BSA addition, and 1:10 RNA dilution. Importantly, in this method-oriented study, we focused on the dust samples only, regardless of the other types of samples, including swabs.

## RESULTS

We conducted a study between December 2021 and April 2022, during the H5N1 high pathogenicity avian influenza epizootic, on a total of 107 French poultry houses. The selected farms were either confirmed HPAIV outbreaks or had a direct epidemiological link with other HPAIV-positive farms. Dust samples were collected using dry wipes rubbed against the walls of the buildings or the feed troughs.

We performed on each sample an RT-qPCR targeting the hemagglutinin and the matrix gene segments, using primers and probes recommended by the World Organization for Animal Health (WOAH), the Food and Agriculture Organization (FAO), and the French Agency for Food, Environmental, and Occupational Health and Safety (ANSES) for the detection of Eurasian H5 HPAIVs (17, 18). We compared the addition of BSA (at a final concentration of 1 µg/µL) to the RT-qPCR reaction mix and the 1:10 dilution of template RNA. Of the 107 dust samples, 19.6% (*n* = 21) and 16.8% (*n* = 18) were, respectively, HA and M positive with the control protocol (undiluted RNA, no BSA), 26.2% (*n* = 28) and 25.2% (*n* = 27) were, respectively, HA and M positive with the BSA protocol, and 15.9% (*n* = 17) and 14.0% (*n* = 15) were, respectively, HA and M positive with the dilution protocol. All HA and M positive samples in the control protocol were also positive with the addition of BSA and in the dilution protocols. All HA and M positive samples from the dilution protocol were also positive with the BSA protocol. More importantly, 5.6% (*n* = 6) and 6.5% (*n* = 7) samples were, respectively, HA and M positive only under the BSA protocol (Table 1). Detailed results are available in Table S1.

**TABLE 1:** Summary of HA and M RT-qPCR testing results per testing combination

HA				M			
**Control**	BSA	Dilution	*n*	Control	BSA	Dilution	*n*
	-	-	79	-	-	-	81
-	-	+	0	-	-	+	0
-	+	-	6	-	+	-	7
-	+	+	1	-	+	+	1
+	-	-	0	+	-	-	0
+	-	+	0	+	-	+	0
+	+	-	5	+	+	-	4
+	+	+	16	+	+	+	14

**FIG 1:**
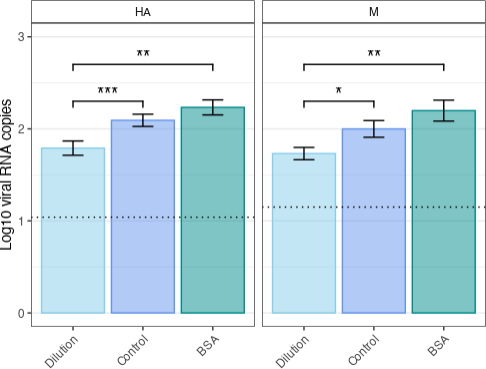
BSA addition is not detrimental to the RT-qPCR reaction. We quantified HA and M RNA levels by RT-qPCR, comparing three protocols (control, 100µg/mL BSA, 1:10 dilution). While diluting the RNA resulted in a statistically significant decrease of viral RNA copy numbers for both HA and M, BSA addition was not detrimental to the PCR reaction, regardless of whether inhibitors were present. *, *p* < 0.05; **, *p* < 0.01. Statistical analysis: two-tailed Mann-Whitney test. Results are expressed as means ± standard error of the mean (SEM). The dotted line represents the limit of detection.

BSA addition was not detrimental to the RT-qPCR reaction. On the contrary, we observed a slight but not statistically significant increase in viral RNA copy numbers compared to the control protocol. As expected, 1:10 dilution of RNAs resulted in a statistically significant decrease in viral RNA copy numbers compared with control conditions and the addition of BSA, for both the HA and M genes (Fig. 1).

To further characterize the effect of BSA, we spiked nine randomly selected negative dust samples with increasing amounts of H5N1 virus, ranging from 10^4^ 50% tissue culture infectious dose (TCID_50_) to 10^7^ TCID_50_, before performing RNA extraction and RT-qPCR (Table S2). When dilutions were made in phosphate-buffered saline (PBS), all samples were positive. Two dust samples became negative for dilutions above 10^5.5^ TCID_50_, while the addition of BSA allowed detection up to 10^4.5^ TCID_50_. Two dust samples became negative for dilutions above 10^5.5^ TCID_50_, while the addition of BSA allowed detection up to 10^5^ TCID_50_. Another became negative from 10^5^ TCID_50_ but was positive up to 10^4.5^ TCID_50_ with BSA. Finally, one sample became negative beyond 10^7^ TCID_50_, and remained positive up to 10^5.5^ TCID_50_ with BSA—we believe this sample was highly loaded with inhibitors. The addition of BSA had no effect on the three samples, suggesting that they did not contain significant amounts of inhibitors. These findings logically suggest that the benefit of BSA depends on both the amount of inhibitors and the initial amount of virus, with a maximum effect at low virus concentrations.

Then, we evaluated the sensitivity of each protocol, specificity, and prevalence using four Bayesian latent class models. For both HA and M, the model with the lowest deviance information criterion (DIC) included conditional dependence between the control and dilution protocols in positive samples (DIC = 20.2). As no other models were within two points of difference in DIC from the best-fit model, this model was selected for inferring the parameter estimates of the proportion of infected farms and the sensitivity of each protocol.

**FIG 2:**
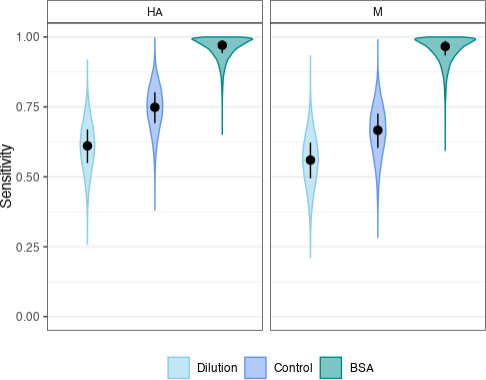
Violin plots of estimated sensitivity of each testing protocol by the best model. The black dot inside each violin indicates the median value, with the bar indicating the interquartile range (IQR). For HA RNA detection, the control protocol resulted in an estimated median sensitivity of 0.75 (IQR 0.69–0.80), the BSA protocol a median sensitivity of 0.97 (IQR 0.94–0.99), and the dilution protocol a median sensitivity of 0.61 (IQR 0.55–0.67). For M RNA detection, the estimated median sensitivity of the control protocol was 0.67 (IQR 0.6–0.73), the BSA protocol 0.97 (IQR 0.93–0.99), and the dilution protocol 0.56 (IQR 0.49–0.62). The best model for both HA and M RNA detection was the one assuming positive conditional dependence between the control and dilution protocols.

As illustrated in Fig. 2, for HA RNA detection, the estimated sensitivity of the BSA protocol was the highest at 0.97 (95% credible interval (CrI) 0.85–1.0), followed by that of the control protocol at 0.75 (CrI 0.57–0.89), and that of the dilution protocol at 0.61 (CrI 0.43–0.77). The estimated specificity of the tests was 1.0 (CrI 0.98–1), the proportion of infected farms among the 107 sampled farms was estimated at 0.27 (0.19–0.36), and the covariance parameter was estimated at 0.09 (CrI 0.02–0.16). For M detection, again the BSA protocol had the highest sensitivity at 0.97 (CrI 0.83–1), followed by the control protocol sensitivity at 0.67 (CrI 0.47–0.83), and lastly the dilution protocol at 0.56 (0.37–0.73). The estimated specificity for M detection was the same as HA detection (1.0 [CrI 0.98–1]). The proportion of infected farms by M detection was estimated at 0.26 (CrI 0.18–0.35), and the covariance parameter was estimated at 0.12 (0.05–0.19).

## DISCUSSION

The relevance of environmental sampling for viral surveillance is increasingly considered and has been emphasized during the COVID-19 pandemic with wastewater-based surveillance, which provided very valuable information, in complement to individual-based PCR testing. It also raised the issue of sewage samples’ RT-qPCR inhibition and the need for optimization and controls in dedicated protocols (19). Dust sampling from walls or equipment located high up in livestock buildings theoretically allows the least possible sampling of feces, litter, or feed, which are known to contain PCR or RT-qPCR inhibitors, often in large amounts (20, 21). In practice, although the sensitivity of dust PCR testing for the detection of influenza viruses is comparable to that of oropharyngeal or tracheal swabs (5), dust can still contain enough inhibitors to disrupt DNA polymerization (22).

For matrices with high levels of amplification inhibitors, dilution of samples or extracted nucleic acids is a widely applied method, since it automatically results in an amplification inhibitors dilution, a 10-fold dilution being commonly used (23, 24). Unfortunately, it usually results in a decrease in sensitivity (12). The addition of substances capable of lifting Rt-qPCR inhibition is therefore a particularly interesting option. Our results reveal that the addition of BSA to the RT-qPCR mix increases its sensitivity. Albumin is a protein capable of binding to numerous compounds: one of BSA’s mechanisms of action could be to bind to inhibitory molecules, thereby inactivating them (25). Other compounds, such as single-stranded DNA binding T4 gene 32 protein (GP32), act in a similar way (12, 25). It should be noted that while BSA addition is beneficial in samples containing melanin, soil, or feces-derived inhibitors, it is not effective against several molecules, such as collagen, bile salts, or bilirubin (12).

The principal limitation of our study is that we did not take cloacal and/or tracheal swabs on poultry farms, thus preventing us from directly comparing dust sampling with swabs. However, this comparison was carried out in a previous study, on a smaller number of poultry houses, showing that RT-qPCR from dust was an equivalent alternative regarding sensitivity (5).

Dust sampling protocols for HPAIVs monitoring are set to become increasingly important in France and Europe, with the European Union imposing stringent monitoring protocols as a prerequisite for the roll-out of vaccination of poultry against H5Nx viruses (9). Such protocols are still to be standardized and rigorously validated, and they can be optimized at various steps. Firstly, by following standardized wiping protocols, covering surfaces—on walls and material—in the poultry house. Secondly, by limiting sample contamination by feces or feed particles. Thirdly, by adapting the technology used to extract the RNA. From one RNA extraction method to another, RNA purity and inhibitor quantities can vary: for example, RNA extracted from magnetic bead-based systems can contain fewer inhibitors than those extracted from column-based systems (26, 27). Finally, RT-qPCR sensitivity can be enhanced by adding inhibition-releasing molecules to the reaction mix.

Among these potential additives, BSA is an inexpensive, non-toxic, and readily available reagent, which makes it easy to use. It could therefore be implemented routinely in dust monitoring RT-qPCR protocols. To conclude, this study is one illustration of the possible analytic improvements that will likely increase further the value of environmental sampling in HPAIV surveillance in the near future.

## MATERIALS AND METHODS

### Dust sampling

Between December 2021 and April 2022, we selected a total of 107 poultry farms, either HPAI-positive as confirmed by official analyses or in close vicinity with HPAI-infected farms, in communes (administrative units) heavily affected by HPAIV epizootics. All samples were collected under the supervision of the French Official Veterinary Services and in compliance with the French legislation on notifiable diseases. In each poultry house, surface dust was collected using dry wipes of approximately 900 cm^2^, rubbed on the buildings’ walls or feeders. Wipes were shipped to the National Veterinary School of Toulouse (France) and stored at 4°C for up to 48 h before being processed. Since other types of samples were not systematically collected in the 107 poultry houses included, we focused only on the dust samples in this study.

### Wipes processing

Twenty milliliter of PBS was added directly to the wipes transport bags. After mixing by hand massage for 2–3 min, the dust solution was collected and aliquoted into 1.5 mL centrifuge tubes, and stored frozen at −80°C, awaiting further analysis.

### Dust spiking with H5N1 virus

We randomly selected 9 HA and M RT-qPCR-negative dusts. We performed 10-fold serial dilutions of the virus in these dust, using the A/Mule duck/France/21348/2021(H5N1) strain previously propagated and titrated on embryonated eggs, ranging from 10^7^ TCID_50_ to 10^4^ TCID_50_. Experiments were performed in biosafety level 3 laboratories.

### RNA extraction and RT-qPCR

Viral RNA was extracted from 100 µL of dust solution using the magnetic bead-based ID Gene Mag Fast Extraction Kit and an IDEAL-96 automated platform, according to the manufacturer’s instructions (Innovative Diagnostics, Grabel, France).

Influenza nucleic acid load was determined by RT-qPCR, using primers and probes recommended by the WOAH, the FAO for the detection of Eurasian H5 HPAIVs and the ANSES (17, 18). Primers and probes sequences are available in Table 2. Briefly, RT-qPCR was performed in 96-well plates in a final volume of 20 µL using a LightCycler 96 system (Roche, Mannheim, Germany). Mixes were prepared according to the manufacturer’s instructions (QuantiNova Probe RT-PCR, Qiagen, Canada) with 4 µL of cDNA and a final concentration of 0.8 µM of each primer and 0.5 µM of the probe. Three protocols were tested: the use of undiluted RNA with or without BSA (at a final concentration of 1 µg/µL), and the use of 1:10 water-diluted RNA. All RT-qPCR reactions were performed in duplicates.

**TABLE 2:** Primers and probe used for RT-qPCR

Target	Name	Sequence (5’ to 3’)	Reference
H5	AIV H5 LH1	ACATATGACTACCCACARTATTCAG	(17, 18)
	AIV H5 RH1	AGACCAGCTAYCATG	
	AIV H5-PRO	TCWACAGTGGCGAGTTCCCTAGCA	
M	AIV M1 u25	AGATGAGTCTTCTAACCGAGGTCG	(18)
	AIV M1 r124	TGCAAAAACATCTTCAAGTCTCTG	
	IPC M	TACGGGGCA AGTGCAATAGAGG	

### Latent class analysis

The sensitivity of the three testing protocols, for both HA and M genes, was estimated jointly through Bayesian latent class analysis (BLCA), an established paradigm endorsed by the WOAH for evaluating test accuracy when the true epidemiological status of tested individuals is unknown (28 – 31). Here, Markov Chain Monte Carlo methods were used to estimate the proportion of infected farms in the sample, testing protocol sensitivity, and specificity. The explicit steps of BLCA have been thoroughly described elsewhere (28, 30). By using Bayesian methods, we eluded sample size power issues, as we did not perform null-hypothesis significance testing (32).

In comparing three tests applied to a single population, there are a total of eight test result combinations. The distribution of the observed frequency of the eight outcome combinations was assumed to follow a multinomial distribution defined by the number of sampled farms (*N* = 107) and the eight probability combinations that can be expressed as a function of the proportion of infected farms the sensitivity and the specificity of each of the three different testing protocols. In our case, given that the primers, the probe, and the templates were the same for each testing protocol, we assumed that the three testing protocols had the same specificity. To test for pairwise conditional positive dependence between testing protocols, three additional models—one for each pairwise combination (i.e., positive dependence between the control and BSA protocols, the control and dilution protocols, and the BSA and dilution protocols) were constructed by including a covariance parameter as established in (33, 34).

Uninformative priors between 0 and 1 were assumed for the prevalence and the three sensitivity parameters. As RT-qPCR is established as a highly specific test (35), the specificity prior was defined through a beta distribution with a median of 98% and a fifth percentile of 80% and given by Beta(11.5, 0.5) (36).

Three chains of 100,000 iterations each were run, allowing for a burn-in of 5,000 iterations, and then thinned to every hundredth sample. Chain convergence was confirmed visually through trace plots and quantitatively via verifying a Gelman-Rubin statistic <1.1, and stability of the limits of the 95% highest density interval (HDI) estimates was confirmed by ensuring effective sample sizes for each parameter over 10,000 (37, 38). Pairwise conditional dependence between testing protocols was assessed by comparing the DIC between the four models, with the best model defined as the most parsimonious model (i.e., the model with the lowest number of parameters) with a DIC value at less than two points from the model with the lowest DIC (36, 39). If no other model is within the two-point limit, the model with the lowest DIC is selected.

Modeling and analysis were performed through the JAGS library via the Rjags package version 4.12 (40) in R version 4.1.3 “One Push-Up” (41).

## SUPPLEMENTAL MATERIAL

Table S1 spectrum.03055-23-s0001.xlsx Viral RNA levels from dust samples.

Table S2 spectrum.03055-23-s0002.xlsx RT-qPCR results from dust samples spiked with increasing amounts of H5N1 virus.

ASM does not own the copyrights to Supplemental Material that may be linked to, or accessed through, an article. The authors have granted ASM a non-exclusive, world-wide license to publish the Supplemental Material files. Please contact the corresponding author directly for reuse.
